# Improved spectrophotometric assay for lytic polysaccharide monooxygenase

**DOI:** 10.1186/s13068-019-1624-3

**Published:** 2019-12-05

**Authors:** Erik Breslmayr, Sarah Daly, Alen Požgajčić, Hucheng Chang, Tonči Rezić, Chris Oostenbrink, Roland Ludwig

**Affiliations:** 1 Biocatalysis and Biosensor Laboratory, Department of Food Science and Technology, BOKU-University of Natural Resources and Life Sciences, Muthgasse 18, 1190 Vienna, Austria; 2 Institute of Molecular Modeling and Simulation, BOKU-University of Natural Resources and Life Sciences, Muthgasse 18, 1190 Vienna, Austria; 3 Department of Biochemical Engineering, Faculty of Food Technology and Biotechnology, University of Zagreb, Pierottijeva 6, 10000 Zagreb, Croatia

**Keywords:** Activity assay, 2,6-Dimethoxyphenol, Hydrocoerulignone, Hydrogen peroxide, Inhibitors, Lytic polysaccharide monooxygenase, Peroxidase activity

## Abstract

**Background:**

The availability of a sensitive and robust activity assay is a prerequisite for efficient enzyme production, purification, and characterization. Here we report on a spectrophotometric assay for lytic polysaccharide monooxygenase (LPMO), which is an advancement of the previously published 2,6-dimethoxyphenol (2,6-DMP)-based LPMO assay. The new assay is based on hydrocoerulignone as substrate and hydrogen peroxide as cosubstrate and aims toward a higher sensitivity at acidic pH and a more reliable detection of LPMO in complex matrices like culture media.

**Results:**

An LPMO activity assay following the colorimetric oxidation of hydrocoerulignone to coerulignone was developed. This peroxidase activity of LPMO in the presence of hydrogen peroxide can be detected in various buffers between pH 4–8. By reducing the substrate and cosubstrate concentration, the assay has been optimized for minimal autoxidation and enzyme deactivation while maintaining sensitivity. Finally, the optimized and validated LPMO assay was used to follow the recombinant expression of an LPMO in *Pichia pastoris* and to screen for interfering substances in fermentation media suppressing the assayed reaction.

**Conclusions:**

The biphenol hydrocoerulignone is a better substrate for LPMO than the monophenol 2,6-DMP, because of a ~ 30 times lower apparent *K*_M_ value and a 160 mV lower oxidation potential. This greatly increases the measured LPMO activity when using hydrocoerulignone instead of 2,6-DMP under otherwise similar assay conditions. The improved activity allows the adaptation of the LPMO assay toward a higher sensitivity, different buffers and pH values, more stable assay conditions or to overcome low concentrations of inhibiting substances. The developed assay protocol and optimization guidelines increase the adaptability and applicability of the hydrocoerulignone assay for the production, purification, and characterization of LPMOs.
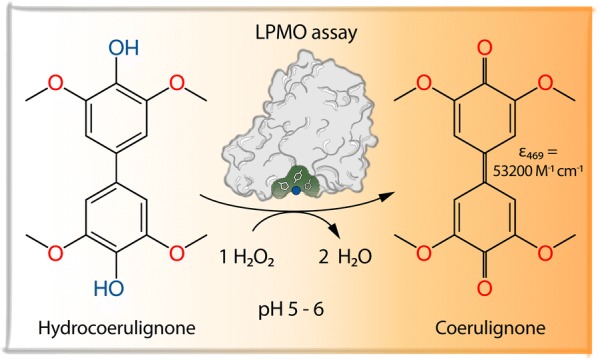

## Background

The number of characterized lytic polysaccharide monooxygenases (LPMO) has been steadily increasing over the last years and resulted in the discovery of new substrate specificities and classification of LPMOs by the Carbohydrate-Active enZYme (CAZy) database or the Enzyme Commission (EC). Currently, known LPMO activities are allocated to CAZy auxiliary activities AA9 [EC 1.14.99.54 lytic cellulose monooxygenase (C1-hydroxylating) and EC 1.14.99.56 lytic cellulose monooxygenase (C4-dehydrogenating)], AA10 (EC 1.14.99.53, lytic chitin monooxygenase), AA11, AA13 (EC 1.14.99.55 lytic starch monooxygenase), AA14, AA15, and AA16 [1–4] all involved in the degradation of polysaccharides [5]. The search for novel LPMOs is driven by the need to improve industrial biomass utilization by reducing the time of hydrolysis and increasing the specificity of the depolymerization process. Before assessing the properties of a newly discovered LPMO, it has to be recombinantly produced and purified. A fast and sensitive assay is therefore a necessity to optimize fermentation and purification protocols or to simplify its biochemical characterization, e.g., deactivation studies. Ideally, such an assay should detect LPMOs from various classes without being limited by the use of class-specific polysaccharide substrates.

Based on a study, in which Bissaro et al. [6] demonstrated that H_2_O_2_ is a cosubstrate of LPMOs, we previously developed a colorimetric assay that employs LPMO’s active site copper center in a peroxidase-like reaction to convert 2,6-DMP into the highly colored product coerulignone [7]. The conversion of 2,6-DMP by LPMO, a small phenolic compound occurring in lignin, is not unexpected considering that LPMOs can also oxidize similar lignin degradation products to obtain the necessary electron for its active site copper activation [8–10]. The formation of coerulignone involves two steps and starts with the oxidation of two 2,6-DMP molecules, which spontaneously dimerize to a dimer (hydrocoerulignone) and cannot be followed in the visible range. In the second step, the formed hydrocoerulignone molecule is oxidized to the chromogenic compound coerulignone. The stoichiometry of the reaction 2,6-DMP → hydrocoerulignone → coerulignone is 2:1:1, and in total, two H_2_O_2_ molecules are consumed. The molar absorption coefficient of coerulignone is *ε*_469_ = 53,200 M^−1^ cm^−1^, which makes this reaction product suitable for a sensitive LPMO assay. However, 2,6-DMP is a poor substrate for LPMO because of two reasons: (1) the apparent *K*_M_ value of LPMO for the monophenol 2,6-DMP is very high (~ 100 mM, [7])⁠ and (2) the oxidative potential of 2,6-DMP is close to the midpoint potential of LPMOs (− 50 to + 121 mV vs. Ag|AgCl, [11]), which reduces the driving force of the reaction, especially at acidic pH and low 2,6-DMP concentrations [7]. A similar effect on the efficiency of a reductant in regard to redox potential and pH has been observed for 2,3-dihydroxybenzoic acid [12].

The 2,6-DMP assay has been used in different studies to detect LPMOs peroxidase activity [13–15] to compare different LPMO fractions during purification [16] or to study the thermal stability of an LPMO [17]. However, some users have indicated that the 2,6-DMP assay does not work for certain LPMOs. We therefore want to point out that, although we have found this peroxidase activity with all LPMOs we tested so far, this is no guarantee that all LPMOs can be screened or detected. Also the wish for an easier, more sensitive and more robust assay was addressed. We observed in preliminary studies that the reaction intermediate hydrocoerulignone (also a compound derived from lignin) can replace 2,6-DMP as a substrate in the LPMO activity assay. The oxidation of 2,6-DMP to hydrocoerulignone was found to be the rate-limiting step in the reaction and the rate of hydrocoerulignone conversion was ~ 15 times faster compared to 2,6-DMP. An activity assay for LPMO employing hydrocoerulignone should therefore be suitable to detect LPMO activity at a lower enzyme concentration and at slightly acidic, more physiological pH conditions (e.g., pH ~ 5.5 in wood cell walls, [18]).

**Fig. 1:**
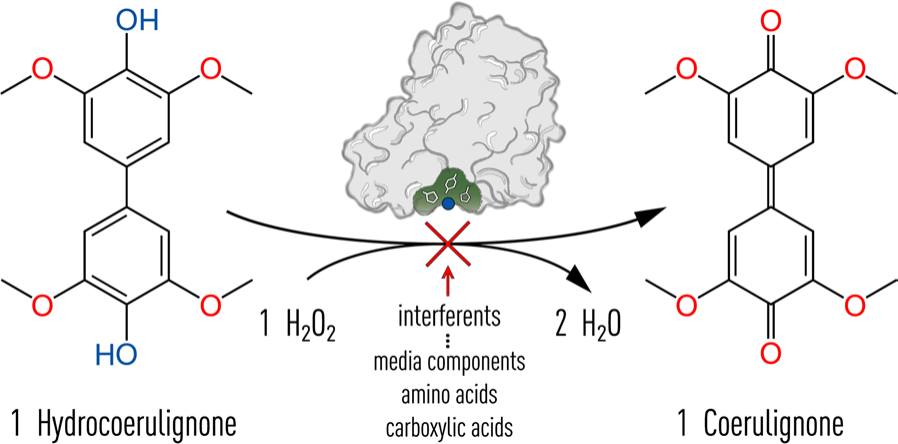
Reaction scheme of hydrocoerulignone oxidation by LPMO. Hydrocoerulignone is oxidized at the expense of H_2_O_2_ to form the chromogenic product coerulignone (*ε*_469_ = 53,200 M^−1^ cm^−1^) and water. The stoichiometry of this reaction is 1. The reaction can be inhibited by different interfering compounds which are indicated below the red arrow

The assayed LPMO peroxidase-like reaction depends on the initial reduction of the LPMO copper active site from its resting state Cu(II) to Cu(I) by hydrocoerulignone and the subsequent binding of H_2_O_2_ to form the chromogenic compound coerulignone with a stoichiometry of 1:1 (Fig. 1). However, it is obvious that the surface exposed copper active site is not protected from metal-chelating molecules. When testing LPMO activity with 2,6-DMP, we observed inhibition by different buffer species, e.g., citric acid or histidine. Therefore, different media components or substances secreted by fungi, like amino acids, salts/ions, and carboxylic acids were screened for an inhibitory effect on the assay. Several rounds of optimization were performed on the assay to find robust and sensitive assay parameters and conditions. The selection of the assay buffer, its concentration, and pH, the substrate and cosubstrate concentration, as well as the applied LPMO concentration/activity is described in the following sections before a protocol of the developed assay is presented. Insights into LPMO’s peroxidase activity are summarized in a guideline on how to modify the assay for maximum sensitivity or how to troubleshoot potential problems.

## Results

### Assay preparations and initial measurements

The stoichiometry of the assayed reaction (Eq. 1) has been established in previous publications [7, 19],

1 $$1 {\text{ hydrocoerulignone}} + 1 {\text{ H}}_{ 2} {\text{O}}_{ 2} \to 1 {\text{ coerulignone }} + {\text{ 2 H}}_{ 2} {\text{O}}$$

where hydrocoerulignone is the first reaction product of the LPMO catalyzed conversion of 2,6-dimethoxyphenol (2,6-DMP) to the colorimetric product coerulignone (Eq. 2),

2 $${2}\;{2},6{\text{-DMP}} + 1\,{{\text{H}}_{2}}{{\text{O}}_{2}} \to 1\,{\text{hydrocoerulignone}} + 2\,{{\text{H}}_{2}}{\text{O}}$$

which is followed by the reaction given in Eq. 1 to result in the overall reaction (Eq. 3).

3 $$2\;2,6{\text{-DMP}} + 2\, {{\text{H}}_2}{{\text{O}}_2} \to 1\,{\text{coerulignone}} + 4\,{{\text{H}}_2}{\text{O}}$$

The molar absorption coefficient of hydrocoerulignone at 280 nm was determined to be 16,260 M^−1^ cm^−1^ (Additional file 1). The reaction rate of LPMO with hydrocoerulignone is ~ 15 times faster compared to 2,6-DMP [7]. Reasons could be a better binding to the active site of LPMO, which is demonstrated by a lower apparent *K*_M_ value for hydrocoerulignone than for 2,6-DMP (data shown below), or a more favorable, lower redox potential.

**Fig. 2:**
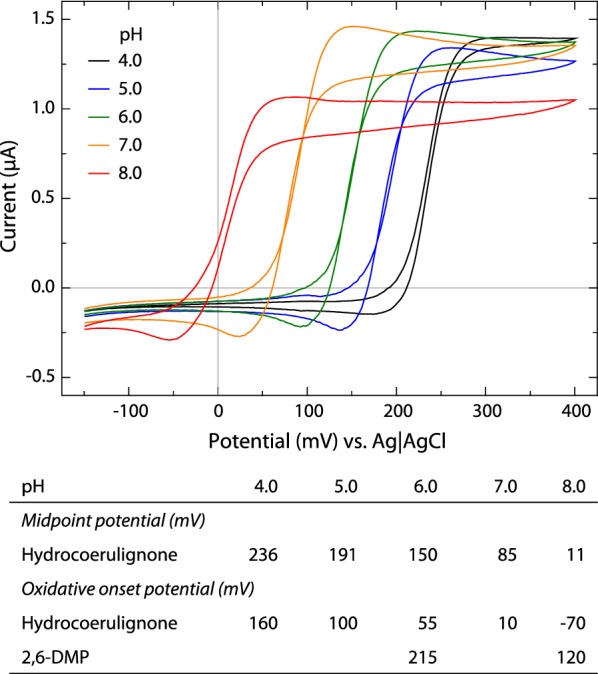
Determination of the hydrocoerulignone midpoint potentials and oxidative onset potentials by cyclic voltammetry using 500 µM hydrocoerulignone in 50 mM sodium phosphate buffer between pH 4.0 and 8.0. At pH 7.0 and especially pH 8.0, the autoxidation and polymerization of hydrocoerulignone were observed. The data extracted from the cyclic voltammograms are given in the table below and are compared to data for 2,6-DMP (300 µM) from Breslmayr et al. [7]

The oxidative onset potential of hydrocoerulignone (Fig. 2) is at pH 6.0 about 160 mV lower than that of 2,6-DMP. The pH-dependent increase in the hydrocoerulignone-coerulignone midpoint potential and onset potential follow roughly the Nernst equation, but Δ*E* is smaller than 59 mV below pH 6 and larger above. At pH 6.0 and a 500 µM concentration, the oxidative onset potential of hydrocoerulignone is 55 mV vs. Ag|AgCl. In comparison, at pH 6.0 and a 300 µM concentration, the oxidative onset potential of 2,6-DMP is 215 mV vs. Ag|AgCl [7]. The higher oxidation potentials at acidic pH (e.g., hydrocoerulignone at pH 4.0 is 236 mV vs. Ag|AgCl) might be very close to or exceed the midpoint potential of the active site copper and thereby limit LPMO activity to less acidic pH. The use of the oxidative onset potential for a comparison with 2,6-DMP is necessary as no midpoint potential can be determined for 2,6-DMP. The reason is the non-reversible reaction of 2,6-DMP to hydrocoerulignone and the fast, subsequent reaction of hydrocoerulignone to coerulignone.

### LPMO activity is affected by buffer concentration, denticity, and pH

**Fig. 3:**
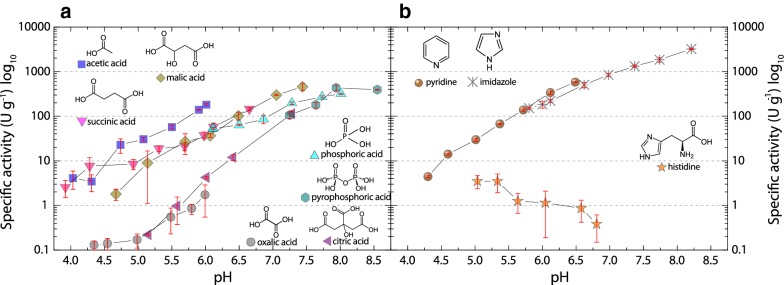
pH profile of *Nc*LPMO9C (0.3 µM) activity on 1000 µM hydrocoerulignone and 100 µM H_2_O_2_ in various buffers. **a** 100 mM carboxylate or phosphate buffers titrated with NaOH and **b** 100 mM cationic pyridine, imidazole, or histidine buffers titrated with HCl. All data are shown as mean values (± SD), from four independent repeats

The pH-dependent activity of LPMO with hydrocoerulignone was measured in different buffers. A general, monotonic increase in activity was observed between pH 4–8. This strong increase in activity, which we correlate to the decrease of the hydrocoerulignone oxidation potential at higher pH values, made it necessary to use a semilogarithmic scale for the pH profile (Fig. 3). Several mono-, di-, and tricarboxylic acids, hydroxycarboxylic acids, and phosphoric acids were used as anionic buffer species as well as pyridine, imidazole as cationic buffer species, and histidine as a zwitterion. The denticity of di- and tricarboxylic acids has a strong influence on the LPMO activity. The measured activity is higher in buffers with a lower equivalent of carboxy or hydroxyl groups and decreases in the following order: acetate > succinate > malate > citrate > oxalate (Fig. 3a). In oxalate and citrate buffer, LPMO shows the lowest activity, which at pH 6.0, is 100-fold and 50-fold lower, respectively. The low activity can be correlated with the chelating properties of bi- or tridentate anions, which potentially bind to the active site copper in LPMO.

Pyridine–HCl and imidazole–HCl as cationic buffers result in LPMO activities as high as in the best anionic buffer sodium acetate (Fig. 3b). The pH profile of the zwitterion histidine is the only one which shows a low, constantly decreasing activity between pH 5.5 and 7.0, which indicates that the deprotonation of its imidazole ring in combination with the carboxy or amine group of histidine inhibits LPMO activity by a stronger binding to the active site copper atom. Even when avoiding bi- and tridentate buffer species, many buffers can be used. However, we suggest a simple sodium acetate buffer for the pH range from pH 4.5–6.0, or an imidazole–HCl buffer for enhanced activity in the pH range from pH 6.0–8.0.

### Determination of steady-state kinetic constants for LPMO

To evaluate the most useful substrate and cosubstrate concentrations in the assay, the apparent kinetic constants of *Nc*LPMO9C for hydrocoerulignone and H_2_O_2_ were determined (Table 1, Additional file 2). Depending on the selected concentration of the cosubstrate H_2_O_2_ (3.18–300 µM) the *K*_M,app_ value for hydrocoerulignone increases from 0.6 to 4.8 mM, respectively. The *K*_M,app_ value for the biphenol hydrocoerulignone measured at a cosubstrate concentration of 100 µM H_2_O_2_ is 3.6 mM, which is much more suitable for the assay than the high *K*_M_ value for the monophenol 2,6-DMP (~ 100 mM) at the same cosubstrate concentration. However, at concentrations above 1 mM hydrocoerulignone, the nonenzymatic oxidation to coerulignone results in an already high reaction background, which reduces the signal-to-noise ratio and prevents kinetic measurements under pseudo-first-order conditions. The highest signal-to-noise ratio was obtained when using 500 µM hydrocoerulignone and 100 µM H_2_O_2_. The ratio of the blank reaction over the LPMO reaction is given in a spider diagram for hydrocoerulignone concentrations between 62.5 and 2000 µM in Additional file 3. For the cosubstrate H_2_O_2_ LPMO shows a much higher affinity. The *K*_M,app_ values for H_2_O_2_ at pH 6.0 are between 1.4 and 7.8 µM depending on the used hydrocoerulignone concentration (Table 1).

**Table 1:** Kinetic constants of *Nc*LPMO9C for H_2_O_2_ and hydrocoerulignone determined in 50 mM sodium phosphate buffer, pH 6.0

H_2_O_2_ catalytic constants			Hydrocoerulignone catalytic constants		
Hydrocoerulignone (µM)	*K*_M,app_ (µM)	*V*_max,app_ (U g^−1^)	H_2_O_2_ (µM)	*K*_M,app_ (µM)	*V*_max,app_ (U g^−1^)
2000	7.8 ± 0.8	194.2 ± 4.2	300	4800 ± 900	644 ± 93
1000	4.3 ± 0.6	118.1 ± 2.8	*100*	*3600* ± *500*	*534* ± *58*
*500*	*4.2* ± *0.5*	*63.5* ± *1.2*	50	2100 ± 200	327 ± 21
250	2.7 ± 0.3	36.4 ± 0.7	25	1700 ± 300	255 ± 28
125	1.7 ± 0.5	19.1 ± 0.6	12.5	2000 ± 400	250 ± 29
62.5	1.4 ± 0.2	12.5 ± 0.2	6.25	1300 ± 400	151 ± 22
			3.18	600 ± 100	71 ± 6

Italiziced data represent the concentration of substrates in the assay

The turnover stability of LPMO in the hydrocoerulignone assay is higher than in the DMP assay. A comparison of the 2,6-DMP assay under standard conditions (50 mM succinate phosphate buffer, pH 7.5, 30 °C, 1000 µM 2,6-DMP, 100 µM H_2_O_2_) with the hydrocoerulignone assay under standard conditions (100 mM sodium acetate buffer, pH 6.0, 30 °C, 500 µM hydrocoerulignone, 100 µM H_2_O_2_) showed that the 2,6-DMP activity of *Nc*LPMO9C decreased noticeable after 5 min in which it produces 4 µM coerulignone per µM of enzyme. Based on the reaction stoichiometry, this results in a total turnover of 8 H_2_O_2_ molecules per enzyme. In contrast, *Nc*LPMO9C in the hydrocoerulignone assay was stable for more than 10 min and produced 61 µM coerulignone per µM of enzyme. This results in a total turnover of 61 H_2_O_2_ molecules, which is ~ 8 times higher than in the 2,6-DMP assay.

The peroxidase activity of six different LPMOs from two different organisms was assayed with hydrocoerulignone. The specific activity of purified enzyme preparations was measured at standard conditions with hydrocoerulignone and 2,6-DMP assay for *Neurospora crassa* LPMO9C, LPMO9E, and LPMO9J and for *Crassicarpon hotsonii* (syn*. Myriococcum thermophilum*) LPMO (gene identifier) Myrth2p4_000359, Myrth2p4_004260, and Myrth2p4_006403 (Table 2). All LPMOs were active with both assays and the determined specific activities show a faster reaction with hydrocoerulignone.

**Table 2:** Specific activities of six different LPMOs from two different organisms

	Specific activity (U g^−1^)	
	Hydrocoerulignone	2,6-DMP
*Neurospora crassa*		
LPMO9C	138 ± 12	31 ± 3
LPMO9E	113 ± 4	22 ± 3
LPMO9J	86 ± 4	13 ± 1
*Crassicarpon hotsonii* (syn. *Myriococcum thermophilum*)		
LPMO (gene identifier) Myrth2p4_000359	120 ± 5	30 ± 1
LPMO (gene identifier) Myrth2p4_004260	137 ± 4	16 ± 1
LPMO (gene identifier) Myrth2p4_006403	110 ± 2	38 ± 2

Measured under standard conditions with 500 µM hydrocoerulignone (100 mM sodium acetate buffer, pH 6.0 containing 100 µM H_2_O_2_) and 2,6-DMP (50 mM sodium phosphate buffer, pH 7.5 containing 100 µM H_2_O_2_). Data expressed as mean values (± SD), from three independent repeats

### Linear range and limit of detection

**Fig. 4:**
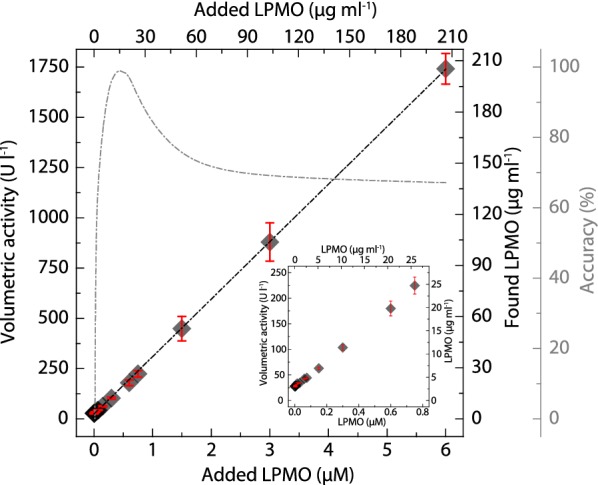
Recovery study for different *Nc*LPMO9C concentrations at pH 6.0. Dark gray diamonds represent the found *Nc*LPMO9C concentration (*Y*-axis) plotted against the added *Nc*LPMO9C concentration (*X*-axis). The activity was determined at 30 °C using 500 µM hydrocoerulignone and 100 µM H_2_O_2_ in 100 mM sodium acetate buffer, pH 6.0. The found *Nc*LPMO9C concentration was calculated from the measured volumetric activity using a specific activity of 138 ± 12 U g^−1^ and the molecular mass of *Nc*LPMO9C (34,400 g mol^−1^). Gray dashed dotted trace shows the concentration range of *Nc*LPMO9C with the highest accuracy taking the standard deviation into account. The inset shows the low concentration range with the highest precision. All data are expressed as mean values (± SD), from four independent repeats

A recovery study was performed to obtain the assay’s limit of detection for LPMO at pH 6.0, a commonly observed pH in fungal cultures (Fig. 4). *Nc*LPMO9C was added to the assay in a concentration between 0.01 and 6.00 µM. The measured rates from the initial 300 s of the reaction are plotted versus the added *Nc*LPMO9C concentrations. The activity was directly proportional to the enzyme concentration. The lower limit of the useful range is defined by the limit of detection (LOD) defining the lower limit of a reliable measurement with respect to the measurement noise occurring from autoxidation of the substrate hydrocoerulignone. The limit of blank (LOB) was determined from 48 measurements exchanging LPMO by buffer. The LOD was determined from 16 different *Nc*LPMO9C concentrations, measured in four, fully randomized technical repeats. Assuming a Gaussian distribution, 95% represents the observed LOB values or LOD low concentration sample values that exceed the defined LOB, respectively. The remaining 5% of blank values are false positive, and only 5% of the low concentration samples will produce values below the LOB. When using 1.645 × SD, no more than 5% of the values should be less than the LOB [20]. The LOD defines the lowest LPMO concentration that can be discriminated from the blank and was determined to be 0.015 µM (0.52 µg ml^−1^) of *Nc*LPMO9C at pH 6.0. When assuming that the average obtained specific LPMO activity of all measurements is 138 U g^−1^ and using the standard deviation of all individual measurements for the estimation, the LPMO concentration range with the highest accuracy can be determined. The highest accuracy, with the lowest standard deviation, is obtained for *Nc*LPMO9C concentration of 0.2–1.2 µM (6–40 µg ml^−1^).

### Monitoring of *Nc*LPMO9C expression

**Fig. 5:**
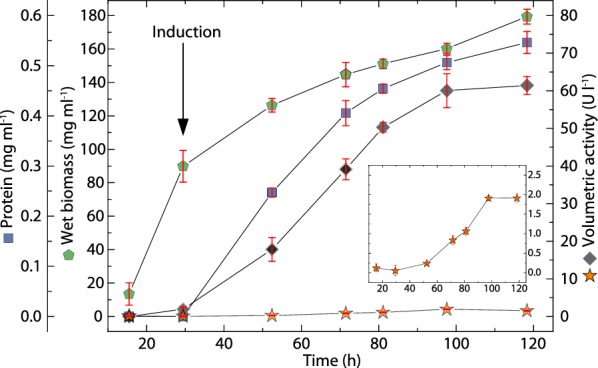
Recombinant expression of *Nc*LPMO9C using *P. pastoris* followed by the hydrocoerulignone assay. LPMO expression was induced after 29 h using methanol. The fermentation medium was supplemented with copper sulfate to reach a final concentration of 100 µM. A sample was immediately taken after copper addition to the medium to obtain a reference. Green pentagons show wet biomass, blue squares show extracellular protein concentration, black diamonds show activity measured with 500 µM hydrocoerulignone as substrate (100 mM sodium acetate buffer, pH 6.0, 20 µl sample volume) and orange stars show activity with 1000 µM 2,6-DMP (50 mM sodium phosphate buffer, pH 7.5, 100 µl sample volume). The activity of 2,6-DMP is magnified in the inset, which has the same units as the *X*-axis and the right *Y*-axis. All activity measurements were performed for 300 s at 30 °C by the addition of 100 µM H_2_O_2_. All data are expressed as mean values (± SD) from three independent measurements

To test the applicability of the LPMO activity assay for monitoring the recombinant expression of LPMO a *P. pastoris* fermentation according to Kittl et al. [21] was performed. We used the hydrocoerulignone assay as described in the protocol below (20 µl sample volume, 100 mM sodium acetate buffer, pH 6.0) and the 2,6-DMP assay (100 µl sample volume, 50 µM sodium phosphate buffer, pH 7.5) to determine the activity of expressed *Nc*LPMO9C in the supernatant (Fig. 5). LPMO expression was induced with methanol and additional CuSO_4_ was added to a final concentration of 100 µM to supplement copper for the active site of LPMO. Directly after the addition of copper a sample was taken to measure its effect on the assay. The presence of copper in the medium gave a negligible response of 1.9 ± 0.6 U l^−1^ (Fig. 5). After induction (29 h after start of the batch phase), the extracellular protein concentration increased over the next 90 h and so did the secreted *Nc*LPMO9C activity. We measured LPMO activity directly from the centrifuged supernatant of the fermentation. The 2,6-DMP assay indicated a low, but measurable volumetric activity when using 100 µl supernatant to increase the LPMO concentration in the assay. The hydrocoerulignone assay gave a much higher volumetric activity with only 20 µl supernatant, which makes the hydrocoerulignone assay a much more sensitive alternative to the 2,6-DMP assay for monitoring the fermentation progress. The measurements showed nonlinearity between the used sample volumes and the determined enzymatic activities, with higher sample volumes resulting in proportionally lower volumetric activities. This was tested by adding purified *Nc*LPMO9C to the fermentation media, which resulted in a 25% lower activity of LPMO measured by the hydrocoerulignone assay (pH 6.0) and a 63% lower activity of LPMO measured by the 2,6-DMP assay (pH 7.5), which indicated an influence of the sample matrix on the assay.

### Screening for inhibiting substances of the LPMO reaction

Similar to the discovered effects of different buffer species, the matrix, e.g., the fermentation medium, can inhibit the reaction. Therefore, we screened for inhibitors that can compromise the detection of LPMO in a fermentation supernatant. For this screening both, the 2,6-DMP assay and the hydrocoerulignone assay were applied in a 100 mM sodium acetate buffer, pH 6.0. Different media components such as yeast extract, yeast nitrogen base (YNB), casein peptone, and meat peptone, which are typically used in fermentation media were tested in two relevant concentrations (1 and 10 g l^−1^; 9 g l^−1^ for YNB, Table 3). Under the same conditions, both assays are similarly affected, resulting in a decreased LPMO peroxidase activity with higher concentrations of the media component. The weakest inhibition was observed for YNB (75% residual activity at 1 g l^−1^) and the strongest inhibition for yeast extract (30–50% residual activity at 1 g l^−1^). The addition of 10 g l^−1^ yeast extract almost fully inhibits the LPMO activity in both assays.

**Table 3:** Effect of media components on *Nc*LPMO9C activity

	Residual activity (%)			
	Hydrocoerulignone		2,6-DMP	
Concentration (g l^−1^)	1.0	10.0	1.0	10.0
Yeast nitrogen base (YNB)^a^	78.8 ± 3.2	29.0 ± 3.9	85.3 ± 3.9	40.1 ± 3.7
Meat peptone	52.6 ± 1.3	23.2 ± 1.5	68.5 ± 5.0	10.9 ± 2.4
Casein peptone	51.8 ± 3.3	12.8 ± 1.0	62.6 ± 2.3	15.6 ± 1.9
Yeast extract	29.9 ± 1.5	13.8 ± 1.5	50.9 ± 1.8	4.4 ± 0.8

Residual activities were calculated based on the concentration of added *Nc*LPMO9C and its determined specific activity (138 U g^−1^ with hydrocoerulignone; 23 U g^−1^ 2,6-DMP). Assay conditions for hydrocoerulignone or 2,6-DMP respectively: 500 or 2000 µM hydrocoerulignone or 2,6-DMP, 0.3 or 2.0 µM *Nc*LPMO9C, 100 µM H_2_O_2_ in 100 mM sodium acetate buffer at pH 6.0 and 30 °C. The pH was measured before and after measurements. Data expressed as mean values (± SD), from three independent repeats

^a^YNB 9 g l^−1^

To specify the media components inhibiting LPMO’s peroxidase activity, specific components of these fermentation media were assayed: all 20 natural amino acids, different cations and anions, and carboxylic acids occurring in the fermentation supernatant or released by yeast or fungi during growth. Amino acids with a terminal carboxylic group on the side chain show a decrease in activity of at least 50%, which is in correspondence with the trend that carboxylic groups inhibit the reaction (Table 4). As mentioned, *Nc*LPMO9C shows negligible activity if histidine at pH 6.0 is present. Also for cysteine no LPMO activity was observed. Aromatic amino acids show the strongest inhibition. Phenylalanine and tryptophan decrease the residual activity to 7 and 3%, respectively, even at a lower concentration than other amino acids (45 mM). Tyrosine, because of its even lower solubility was measured at a 2 mM concentration, which shows still an inhibition of about 10%. Salts/ions have very little or no effect on the LPMO activity except sulfate, which decreases *Nc*LPMO9C activity to 30–39% residual activity (Table 5). This can be interpreted as interaction between the oxyanion and the copper center as already found for phosphate buffers. A strong inhibitory effect was observed for carboxylic acids, especially oxalic acid (Table 6). The bidentate property of oxalic acid could be the reason for the strong inhibitory effect.

**Table 4:** Effect of amino acids on *Nc*LPMO9C activity

	Residual activity (%)	
	Hydrocoerulignone	2,6-DMP
Arg (R)	43.9 ± 0.7	34.0 ± 3.1
His (H)	0.3 ± 0.3	0.1 ± 0.1
Lys (K)	53.5 ± 2.7	36.0 ± 3.7
Asp (D)^a^	39.9 ± 3.3	50.8 ± 1.5
Glu (E)^a^	57.4 ± 2.0	46.5 ± 4.8
Ser (S)	19.1 ± 0.9	34.6 ± 1.2
Thr (T)	13.0 ± 0.5	18.5 ± 1.6
Asn (N)	10.8 ± 0.4	10.6 ± 0.7
Gln (Q)	25.2 ± 0.9	35.9 ± 1.6
Cys (C)	3.7 ± 0.2	0.1 ± 0.1
Gly (G)	49.1 ± 1.2	44.0 ± 3.4
Pro (P)	48.5 ± 1.6	37.1 ± 1.1
Ala (A)	39.9 ± 1.7	62.5 ± 0.3
Val (V)	23.3 ± 2.3	42.4 ± 2.2
Ile (I)	35.5 ± 0.9	47.9 ± 3.2
Leu (L)	38.6 ± 1.1	49.1 ± 0.8
Met (M)	12.0 ± 0.6	21.7 ± 1.7
Phe (F)	7.5 ± 0.4	14.9 ± 0.1
Tyr (Y)^a^	81.5 ± 3.6	91.8 ± 8.3
Trp (W)^a^	3.5 ± 0.4	16.2 ± 2.3

Assays and calculations were performed as indicated in Table 2. Data expressed as mean values (± SD), from three independent repeats

^a^Asp (D), Glu (E), Trp (W) 45 mM; Tyr (Y) 2 mM

**Table 5:** Effect of salts on *Nc*LPMO9C activity

	Residual activity (%)	
	Hydrocoerulignone	2,6-DMP
NaF	65.9 ± 10.0	74.0 ± 2.1
NaCl	105.0 ± 2.6	92.9 ± 0.6
KCl	80.4 ± 0.8	91.6 ± 2.8
MgCl_2_	94.8 ± 5.7	103.5 ± 2.8
CaCl_2_	84.0 ± 1.4	107.2 ± 2.3
NaI	n.m.	86.7 ± 25.3
KBr	84.5 ± 5.5	84.5 ± 6.2
NaNO_3_	82.8 ± 1.2	97.2 ± 2.7
Na_2_SO_4_	30.2 ± 4.5	38.8 ± 3.4

Assays and calculations were performed as indicated in Table 2. Data expressed as mean values (± SD), from three independent repeats

*n.m.* not measured; strong influences on assay

**Table 6:** Effect of carboxylic acids on *Nc*LPMO9C activity

	Specific activity (U g^−1^)					
	Hydrocoerulignone			2,6-DMP		
Concentration (mM)	30	100	300	30	100	300
Formic acid	243 ± 15	186 ± 4	175 ± 15	36 ± 1	31.3 ± 0.3	17 ± 1
Acetic acid	226 ± 17	138 ± 12	69 ± 10	33 ± 2	23 ± 1	13 ± 1
Oxalic acid	4 ± 1	2 ± 1	0.4 ± 0.3	0.8 ± 0.2	0.2 ± 0.1	0.1 ± 0.1
Malonic acid	24 ± 2	9 ± 1	3 ± 1	3.6 ± 0.1	0.7 ± 0.1	0.1 ± 0.1
Succinic acid	84 ± 3	n.m.	n.m.	15.3 ± 0.4	11.8 ± 0.1	10 ± 1
Malic acid	66 ± 7	28 ± 5	12 ± 2	12.6 ± 0.1	5.4 ± 0.2	2.1 ± 0.1
Tartaric acid	106 ± 5	46 ± 3	19.0 ± 0.4	18 ± 1	9 ± 1	3.5 ± 0.3
Maleic acid	55 ± 4	6 ± 3	0.2 ± 0.1	9.8 ± 0.1	4.6 ± 0.1	2.9 ± 0.2
Citric acid	11 ± 1	4 ± 1	0.3 ± 0.2	1.7 ± 0.1	0.5 ± 0.1	0.2 ± 0.1

Assay conditions for hydrocoerulignone or 2,6-DMP, respectively: 500 or 2000 µM hydrocoerulignone or 2,6-DMP, 0.3 or 2.0 µM *Nc*LPMO9C, 100 µM H_2_O_2_ in 30 mM, 100 mM and 300 mM sodium carboxylate buffers at pH 6.0 and 30 °C. The pH was measured before and after measurements. Data expressed as mean values (± SD), of four independent repeats

### General application rules for the assay

**Fig. 6:**
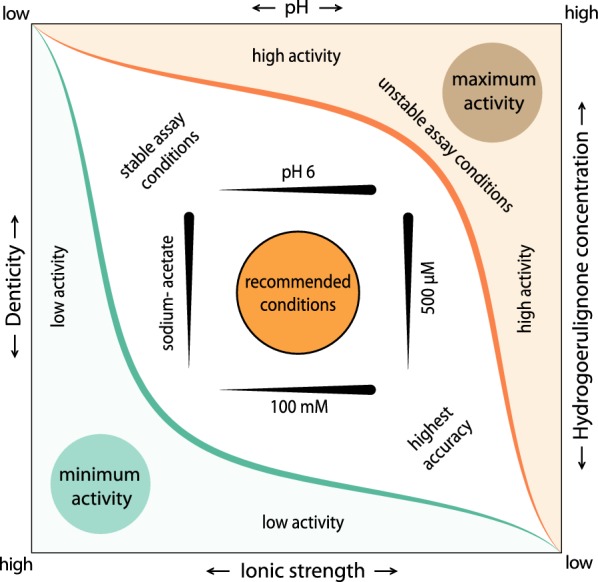
Schematic guideline for the activity assay. Based on all data collected, four major factors were identified to adjust the assay: ionic strength of the buffer, buffer ion denticity, buffer pH and substrate concentration. The green and orange areas correspond to the region lowest and highest LPMO activity, respectively. Black arrows indicate the increase or decrease in activity by adjusting a factor. As a good starting point for the assay, we recommend 100 mM sodium acetate, pH 6.0, 500 µM hydrocoerulignone as chromogenic substrate and 100 µM hydrogen peroxide as cosubstrate. The area around the recommended conditions indicates conditions in which LPMO activity can be detected, but is not optimized for maximum reliability. This region can be used to characterize the LPMO’s behavior under different conditions, e.g., for a pH profile. The area around the recommended conditions indicates conditions in which LPMO activity can be detected, but is not optimized for maximum reliability. This region can be used to characterize the LPMO’s behavior under different conditions, e.g., for a pH profile. We suggest to start with small changes to our recommended conditions to not end up in the green or orange region, where either no activity can be detected or the activity is too high and autoxidation inactivates LPMO too fast

The obtained data were used to optimize the LPMO hydrocoerulignone assay in regard to specificity, accuracy, sensitivity, and robustness to improve its applicability in different matrices. General factors influencing the LPMO peroxidase activity in the hydrocoerulignone assay are shown in Fig. 6. Four factors have to be adjusted to increase the sensitivity and robustness of the assay: (1) Denticity of the buffer species. LPMO activity can be compromised by bi- and tridendate anions. Carboxylic acid groups and the oxy-groups of phosphate and sulfate oxyanions show an inhibitory effect. The monocarboxylic acetate ion compromises LPMO activity very little. (2) Increasing the ionic strength of a buffer generally reduces LPMO activity, and this is observed even for an acetate buffer. (3) A higher pH increases LPMO’s peroxidase activity exponentially, which can be used to measure very low LPMO concentrations at pH 7–8, which enhances the enzymatic activity. (4) An increased concentration of the chromogenic substrate hydrocoerulignone and cosubstrate H_2_O_2_ increases the activity. However, the limits for using very high concentrations is an increased, non-enzymatic autoxidation of hydrocoerulignone at concentrations above 500 µM and an increased deactivation of LPMO activity at H_2_O_2_ concentrations above 100 µM.

The developed assay protocol is a compromise between a non-inhibiting buffer at a close to physiological pH value, and substrate and cosubstrate concentrations providing a good sensitivity without having to consider blank reactions or enzyme deactivation during the assay. However, when using proper controls and a short assay time, the sensitivity of the assay can be boosted by a factor ~ 10–100 using a higher pH, higher hydrocoerulignone and higher H_2_O_2_ concentrations according to Fig. 2 and Table 1. Our recommendation in the following assay protocol is a good starting point for testing LPMO activity, however, we note that other LPMOs can differ from *Nc*LPMO9C and that the assay Factors 1–4 should therefore be considered for each enzyme. In case of multiple measurements required at the same time, the adaptation of the cuvette-based assay to microtiter plates is possible by adjusting the sample and reagent volumes accordingly. However, the shorter and less defined optical pathlength will result in a higher limit of detection and lower sensitivity.

## Discussion

The molar absorption coefficient of coerulignone at 469 nm has been determined to be 53,200 M^−1^ cm^−1^. This high value allows low amounts of produced coerulignone to be detected and ensures a high sensitivity of the assay. The increased activity is not the result of a pH-induced change in the molar absorption coefficient of the chromogenic product coerulignone [7]. The reason to bypass 2,6-DMP in the assay is the faster reaction rate of LPMO with hydrocoerulignone at the physiologically relevant pH around and below pH 6 [10]. The faster reaction rate results from a better binding to the active site of LPMO which is demonstrated by a lower apparent *K*_M_ value for hydrocoerulignone than for 2,6-DMP, but also from a more favorable, lower oxidation potential of hydrocoerulignone. When comparing the difference in the oxidative onset potentials, the 160 mV lower potential of hydrocoerulignone can be recalculated to a ~ 30 kJ mol^−1^ reduction of the Gibbs free energy necessary to overcome the activation energy barrier. Hydrocoerulignone as substrate for LPMO has a very similar behavior when compared to the pH profile published for 2,6-DMP. No major differences are observed. For comparison of both datasets, it has to be noted that pH-dependent activity measurements were using a 1 mM hydrocoerulignone concentration, whereas 25 mM 2,6-DMP was used. The higher 2,6-DMP concentration was necessary to counterbalance the lower activity of LPMO for 2,6-DMP and to speed up the assay. For hydrocoerulignone as substrate, a much lower concentration suffices to obtain an equal specific activity in the assays. The previously published LPMO activity assay based on 2,6-DMP has a LOD of 0.0125 µM (0.43 µg ml^−1^) *Nc*LPMO9C at pH 7.5 [7]. By using hydrocoerulignone, almost the same LOD (0.015 µM, 0.52 µg ml^−1^) can be achieved, however, under the physiologic pH of 6.0, which results for both assays in 50 times lower LOD as for the Amplex red assay [21]. Under steady-state conditions, a direct comparison of the standard assays with hydrocoerulignone and 2,6-DMP showed higher turnover stability for LPMO. One reason is the two times lower H_2_O_2_ turnover of LPMO necessary to convert hydrocoerulignone to coerulignone compared to starting from 2,6-DMP, the other possible reason a lack in 2,6-DMP radical formation. The higher turnover stability and higher rate for LPMO using hydrocoerulignone are advantageous to monitor LPMO activity during fermentations. We could show that by using the hydrocoerulignone assay, the increase in LPMO activity could be correlated with the increase in wet biomass and extracellular protein concentration. However, we recognized lower volumetric activities of purified *Nc*LPMO9C in the fermentation medium, which was confirmed by testing different common media components. Furthermore, several amino acids and carboxylic acids highly inhibit LPMO activity in the assays. Histidine, cysteine, and oxalic acid completely quench the signal. We tested cysteine, which is already known as reductant for LPMO [22, 23] as reductant for coerulignone (data not shown) and conclude that cysteine is, on the one hand, reacting with LPMO and also quickly reducing the final product of the assay. Therefore, LPMO activity cannot be measured in presence of higher cysteine concentrations. For histidine and oxalic acid, a chelating effect is the most obvious conclusion. The bidentate molecule oxalate most potently inhibits the active site of LPMO, possibly through an optimal bidentate binding to the copper. As a general trend, we can conclude that higher denticity and higher ionic strength decreases the LPMO activity in the assay. These kinds of buffer species should be avoided when performing the LPMO activity assays using hydrocoerulignone or 2,6-DMP.

## Conclusions

The hydrocoerulignone-based LPMO activity assay is a fast and easy method to follow recombinant LPMO production and enzyme purification as well as to study enzyme deactivation or substrate binding. When using hydrocoerulignone, LPMO activity can be measured under physiologically relevant, acidic pH conditions, which is an advantage over the 2,6-DMP assay. The lower necessary sample volume reduces the influence of inhibitory matrix compounds and improves the monitoring of LPMO activity during recombinant production and purification. Due to its sensitivity, less protein can be used in biochemical characterization. The inhibition of the assayed LPMO activity by various substances might be a good starting point for further studies on LPMO inhibitors.

## Materials and methods

### Materials and enzymes

All chemicals were of the highest purity grade available and were purchased from Sigma-Aldrich unless stated otherwise. Methanol was purchased from Merck, hydrocoerulignone [3,3′,5,5′-tetramethoxy(1,1′-biphenyl)-4,4′-diol; National Center for Biotechnology Information. PubChem Database. CID = 256604, https://pubchem.ncbi.nlm.nih.gov/compound/256604 (Accessed on 16 Sept. 2019)] from MP Biomedicals (CA, US). Lytic polysaccharide monooxygenases (*Nc*LPMO9C, sequence Accession number EAA36362.1; *Nc*LPMO9E, sequence Accession number EAA26873.1; *Nc*LPMO9J, sequence Accession number CAE81966.1; *Ch*LPMO gene identifier Myrth2p4_000359, Myrth2p4_004260, Myrth2p4_006403) from *Neurospora crassa* and *Crassicarpon hotsonii* (syn: *Myriococcum thermophilum*) were recombinantly expressed in *Pichia pastoris* X-33 according to Kittl et al. [21]. The production in a 5-L bioreactor and chromatographic purification were also performed according to this publication. The purity was verified by SDS-PAGE.

### Measurement of LPMO, protein, and hydrogen peroxide concentration

The concentration of purified LPMO was determined in a 3-mm quartz cuvette from its UV–Vis absorption at 280 nm using the calculated molar absorption coefficient and the molecular mass of *Nc*LPMO9C: *ε*_280_ = 46,910 M^−1^ cm^−1^, 34,300 g mol^−1^; *Nc*LPMO9E: *ε*_280_ = 42,370 M^−1^ cm^−1^, 30,876 g mol^−1^; *Nc*LPMO9J: *ε*_280_ = 47,870 M^−1^ cm^−1^; 32,673 g mol^−1^; *Ch*LPMO (gene identifier) Myrth2p4_000358: *ε*_280_ = 44,140 M^−1^ cm^−1^, 22,515 g mol^−1^; *Ch*LPMO (gene identifier) Myrth2p4_004260: *ε*_280_ = 45,880 M^−1^ cm^−1^, 29,776 g mol^−1^; *Ch*LPMO (gene identifier) Myrth2p4_006403: *ε*_280_ = 39,670 M^−1^ cm^−1^, 32,971 g mol^−1^. The protein concentration in fermentation samples was measured by the method of Bradford using bovine serum albumin for the standard curve [24]. The concentration of H_2_O_2_ in stock solutions was determined in a 10-mm quartz cuvette from the UV–Vis absorption at 240 nm using its molar absorption coefficient of *ε*_240_ = 43.6 M^−1^ cm^−1^.

### Hydrocoerulignone stock solution and molar absorption coefficient

Water, isopropanol, acetonitrile, and DMSO were tested to dissolve hydrocoerulignone from which DMSO showed the best dissolving properties and was selected to prepare the hydrocoerulignone stock solution. To that purpose, hydrocoerulignone was dissolved for 1 h in pure DMSO in a sample rotator. Before further usage the solution was centrifuged to remove minor particles from the supernatant. By measuring the absorbance of various hydrocoerulignone concentrations at pH 6.0 in 50 mM sodium phosphate buffer (remaining DMSO concentration ~ 2%) between 5 and 80 µM the molar absorption coefficient at 280 nm (*ε*_280, hydrocoerulignone_ = 16,260 M^−1^ cm^−1^) was calculated by linear regression (Additional file 1).

### LPMO activity assay

The suggested standard conditions for the LPMO activity assay using hydrocoerulignone are 30 °C, 100 µM H_2_O_2_, 500 µM hydrocoerulignone, 100 mM sodium acetate buffer at pH 6.0 for maximum robustness, sensitivity under physiological conditions and a reaction time of 300 s. For blank reactions, same conditions have to be performed without additions of LPMO and the resulting rate has to be subtracted from the rate with additions of LPMO. One unit of LPMO activity is defined as the conversion of 1 µmol hydrocoerulignone or the formation of 1 µmol coerulignone per min under reaction conditions. The specific activity can be calculated from the slope of the steady-state rate by using the molar absorption coefficient of coerulignone (*ε*_469_ = 53,200 M^−1^ cm^−1^) and the LPMO concentration. For inhibition tests, the 100 mM sodium acetate buffer at pH 6.0 was supplemented with different compounds and the pH measured before and after the measurements.

### LPMO activity assay protocol

Based on the obtained data, we recommend the following basic assay protocol to test the peroxidase activity of LPMO:

- Step 1: Prepare a 106 mM sodium acetate buffer, pH 6.0. The final concentration of the buffer in the cuvette will be 100 mM. Also prepare a 25 mM hydrocoerulignone stock solution in pure DMSO and a 5 mM H_2_O_2_ stock solution in highly pure water. The solutions should not be mixed before the addition to the cuvette and should be used within 12 h.
- Step 2: Take 1 ml of sample from the culture supernatant and centrifuge for 3 min at 6000×*g* to remove cells and other solids. Carefully remove 500 µl of the clear supernatant from the sediment and transfer to a clean vial. If taken from a clear solution, the sample needs no centrifugation and a smaller volume suffices. Store the sample on ice until use.
- Step 3: Transfer 940 µl of buffer, 20 µl of the hydrocoerulignone stock solution and 20 µl of the H_2_O_2_ stock solution into a cuvette and incubate the cuvette for 15 min at 30 °C before continuing with Step 4a and 4b. Due to the autoxidation of hydrocoerulignone a blank reaction should be measured without adding LPMO.
- Step 4a (Reference experiment): Add 20 µl of sodium acetate buffer, pH 6.0 (also used if a dilution of the LPMO sample is done) or a fermentation sample without LPMO and measure the slope of the blank reaction.
- Step 4b (Enzyme assay): Add 20 µl of properly diluted LPMO and measure the slope of the LPMO catalyzed oxidation of hydrocoerulignone. If the LPMO activity is low, a higher sample volume can be used, but the buffer volume and ionic strength have to be adapted accordingly. The increase in absorbance at 469 nm is measured for 300 s at 30 °C and if the blank reaction shows a significant rate, it is subtracted.

Finally, the volumetric LPMO peroxidase activity is calculated from Eq. 4 by using the correct enzyme factor (EF, Eq. 5), which is based on the sample volume, the enzyme dilution, and the molar absorption coefficient of coerulignone (*ε*_469_ = 53.2 mM^−1^ cm^−1^).

4 $${\text{volumetric activity}} \left( {{\text{U}}\;{\text{L}}^{ - 1} } \right) = {\text{rate}} \left( {\min{^{ - 1}} } \right) \times {\text{EF}}$$

5 $${\text{EF}} = \frac{{{\text{total volume}}\;\left( {\text{ml}} \right) \times {\text{dilution factor}}}}{{{\text{sample volume}}\;\left( {\text{ml}} \right) \times {\text{pathlength}}\;\left( {\text{cm}} \right) \times {\text{molar absorption coefficient}}\;\left( {{\text{mM}}^{ - 1} {\text{cm}}^{ - 1} } \right)}}$$

### pH profile of LPMO activity

*Nc*LPMO9C was used to measure enzymatic activity with final concentrations of 100 mM buffer, 100 µM H_2_O_2_, 1000 µM hydrocoerulignone, and 0.3 µM *Nc*LPMO9C. Anionic buffers were titrated with sodium hydroxide and the cationic buffers with hydrochloric acid. All measurements were performed at 30 °C and the change in absorbance at 469 nm was followed in a PerkinElmer Lambda 35 UV/Vis spectrophotometer in quadruplets. Blank reactions were performed for all buffers and pH values to obtain only the LPMO activity.

### Determination of kinetic constants and substrate concentration

Kinetic constants of LPMO were determined in 50 mM sodium phosphate buffer, pH 6.0 using a 0.3 µM *Nc*LPMO9C concentration. All experiments were performed in quadruplets and blank reaction rates were subtracted. The resulting curves were fitted to the Michaelis–Menten equation by nonlinear least-square regression using *SigmaPlot* 12.5 (Systat Software, Chicago, Illinois, USA).

### Cyclic voltammetry

Measurements were performed at 25 °C in a 15-ml electrochemical cell. The system setup involved a rotating disk electrode made from a glassy carbon working electrodes (3 mm in diameter, BASi), an Ag|AgCl (3 M KCl) reference electrode, a platinum counter electrode, an Autolab Rotator (RDE80739), an Autolab controller and an Autolab potentiostat (PBSTAT204). The system was controlled using the NOVA 1.11 program from Autolab. Before measurements, the glassy carbon disc electrode was polished with an aluminum oxide suspension (Buehler; Master Prep Polishing Suspension, 0.05 μm) before each measurement. The rotating disc electrode was set to 250 rpm before starting the cyclic voltammetry measurement by sweeping between − 150 and 400 mV vs. the reference electrode with scan rate of 3 mV s^−1^. For all measurements a 50 mM sodium phosphate buffer was added to the cell. The buffer was left to equilibrate the system for 5 min before hydrocoerulignone was added and mixed for 5 min more to equilibrate before starting the measurement.

### Limit of detection

The limit of detection (LOD) for the LPMO assay with hydrocoerulignone was measured according to Armbruster and Pry [20]. Forty-eight blank reactions without LPMO were measured to calculate the limit of blank (LOB). For the LOD measurement, two independent LPMO dilution series were prepared and measured in a completely randomized measurement scheme, which was prepared by using the RAND function in *Microsoft Excel 2016* (Microsoft Cooperation, Redmond, WA, USA). Quadruplets were measured to calculate the specific activity. To recalculate the *Nc*LPMO9C concentration and to determine the accuracy, which represents the range of *Nc*LPMO9C concentrations with the lowest standard deviation compared with all measurements, the average activity of the most accurate (within ~ 80%) were used.

## Supplementary information

**Additional file 1.** Absorbance of different hydrocoerulignone concentrations at 280 nm for the calculation of the molar absorbance coefficient (*ε*_280_ = 16,260 M^−1^ cm^−1^). LibreOffice_v.6 (Berlin, Germany) was used for a linear regression fit, the calculation of the slope and intercept. Measured in 50 mM sodium phosphate buffer at pH 6.0.

**Additional file 2.** (a) Steady-state kinetic measurements of hydrogen peroxide with 500 μM hydrocoerulignone and (b) of hydrocoerulignone with 100 μM H_2_O_2_. No saturation was achieved for hydrocoerulignone and therefore the kinetic constants for H_2_O_2_ were determined under apparent, non-pseudo-first-order conditions.

**Additional file 3.** Ratio of blank reactions and reaction rates with LPMO of different hydrocoerulignone and H_2_O_2_ concentrations. Grey area, blank reaction lower than 20% of total reaction rate. Measured in 50 mM sodium phosphate buffer at pH 6.0 with *Nc*LPMO9c at 30 °C.

## Data Availability

The datasets used and/or analyzed during the current study are available from the corresponding author on reasonable request.

## Abbreviations

2,6-DMP: 2,6-dimethoxyphenol
hydrocoerulignone: 3,3′,5,5′-tetramethoxy(1,1′-biphenyl)-4,4′-diol
coerulignone: 3,3′,5,5′-tetramethoxy-4,4′-diphenoquinone
DMSO: dimethylsulfoxide
GMC: glucose–methanol–choline
H_2_O_2_: hydrogen peroxide
LOB: limit of blank
LOD: limit of detection
LPMO: lytic polysaccharide monooxygenase
SD: standard deviation
YNB: yeast nitrogen base
