# Supplementary material for: Improved spectrophotometric assay for lytic polysaccharide monooxygenase
Source: Biotechnol Biofuels. 2019 Dec 5;12:283. doi: 10.1186/s13068-019-1624-3 (PMC6894463; doi:10.1186/s13068-019-1624-3)
Supplement: Supplementary file 1 — Additional file 1. Absorbance of different hydrocoerulignone concentrations at 280 nm for the calculation of the molar absorbance coefficient (ε280 = 16,260 M−1 cm−1). LibreOffice_v.6 (Berlin, Germany) was used for a linear regression fit, the calculation of the slope and intercept. Measured in 50 mM sodium phosphate buffer at pH 6.0. [file 13068_2019_1624_MOESM1_ESM.pdf]

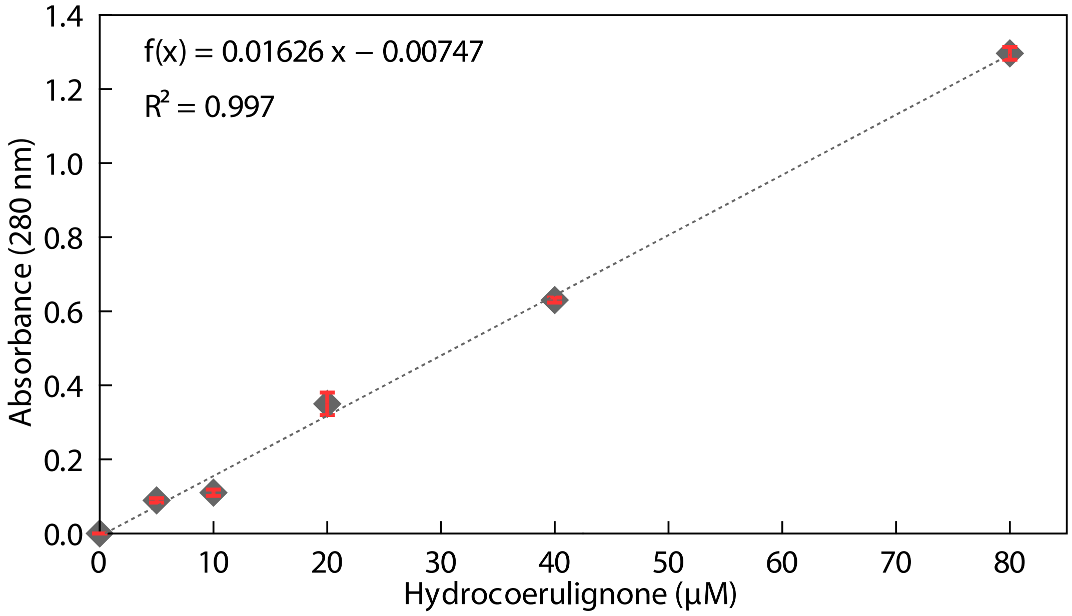

Additional file 1. Absorbance of different hydrocoerulignone concentrations at 280 nm for the calculation of the molar absorbance coefficient (ε_{280} = 16260 M^{-1} cm^{-1}). LibreOffice_v.6 (Berlin, Germany) was used for a linear regression fit, the calculation of the slope and intercept. Measured in 50 mM sodium-phosphate buffer at pH 6.0.
