# Supplementary material for: Improved spectrophotometric assay for lytic polysaccharide monooxygenase
Source: Biotechnol Biofuels. 2019 Dec 5;12:283. doi: 10.1186/s13068-019-1624-3 (PMC6894463; doi:10.1186/s13068-019-1624-3)
Supplement: Supplementary file 2 — Additional file 2. (a) Steady-state kinetic measurements of hydrogen peroxide with 500 μM hydrocoerulignone and (b) of hydrocoerulignone with 100 μM H2O2. No saturation was achieved for hydrocoerulignone and therefore the kinetic constants for H2O2 were determined under apparent, non-pseudo-first-order conditions. [file 13068_2019_1624_MOESM2_ESM.pdf]

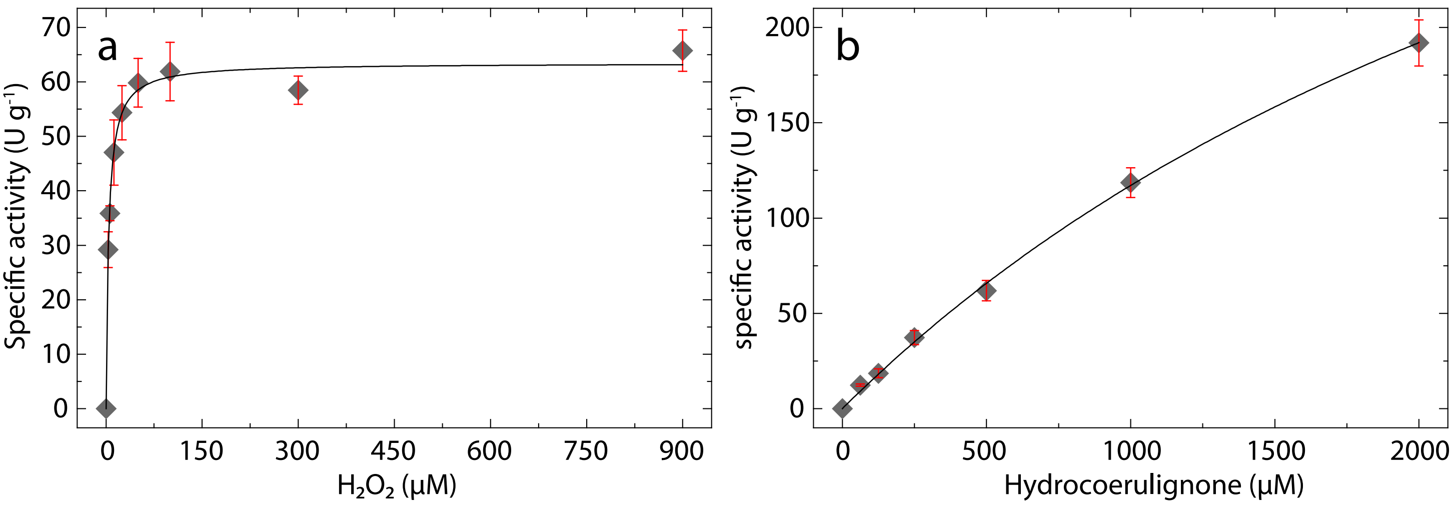

Additional file 2. (a) Steady-state kinetic measurements of hydrogen peroxide with 500 µM hydrocoerulignone and (b) of hydrocoerulignone with 100 µM H_2O_2. No saturation was achieved for hydrocoerulignone and therefore the kinetic constants for H_2O_2 were determined under apparent, non-pseudo-first-order conditions.
