# Supplementary material for: Improved spectrophotometric assay for lytic polysaccharide monooxygenase
Source: Biotechnol Biofuels. 2019 Dec 5;12:283. doi: 10.1186/s13068-019-1624-3 (PMC6894463; doi:10.1186/s13068-019-1624-3)
Supplement: Supplementary file 3 — Additional file 3. Ratio of blank reactions and reaction rates with LPMO of different hydrocoerulignone and H2O2 concentrations. Grey area, blank reaction lower than 20% of total reaction rate. Measured in 50 mM sodium phosphate buffer at pH 6.0 with NcLPMO9c at 30 °C. [file 13068_2019_1624_MOESM3_ESM.pdf]

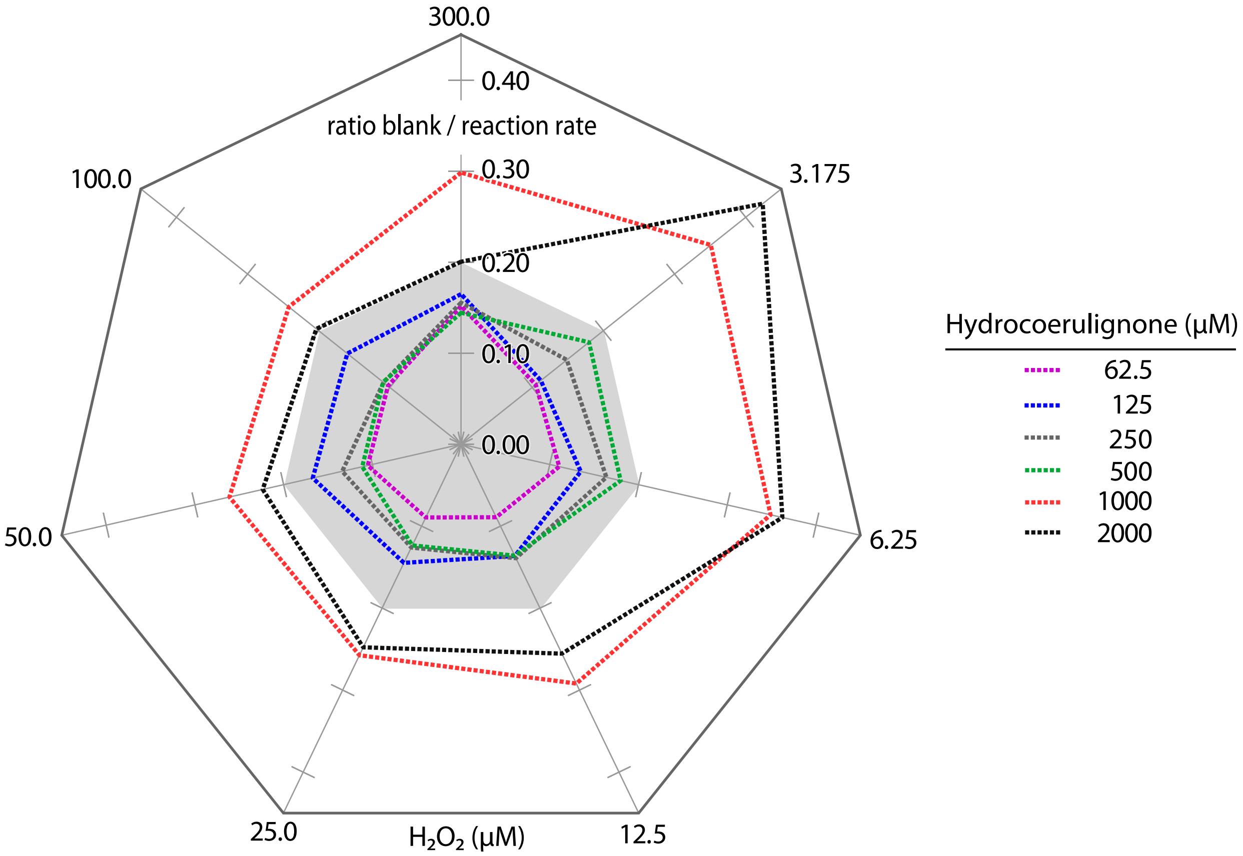

Additional file 3. Ratio of blank reactions and reaction rates with LPMO of different hydrocoerulignone and H_2O_2 concentrations. Grey area, blank reaction lower than 20% of total reaction rate. Measured in 50 mM sodium-phosphate buffer at pH 6.0 with *Nc*LPMO9c at 30°C.
